# Protocol for a quasi-experimental study of the effectiveness and cost-effectiveness of mother and baby units compared with general psychiatric inpatient wards and crisis resolution team services (The ESMI study) in the provision of care for women in the postpartum period

**DOI:** 10.1136/bmjopen-2018-025906

**Published:** 2019-03-23

**Authors:** Kylee Trevillion, Rebekah Shallcross, Elizabeth Ryan, Margaret Heslin, Andrew Pickles, Sarah Byford, Ian Jones, Sonia Johnson, Susan Pawlby, Nicky Stanley, Diana Rose, Gertrude Seneviratne, Angelika Wieck, Stacey Jennings, Laura Potts, Kathryn M Abel, Louise M Howard

**Affiliations:** 1 Section of Women’s Mental Health, Institute of Psychiatry, Psychology & Neuroscience at King’s College London, London, UK; 2 Centre for Academic Primary Care, University of Bristol Medical School, Bristol, UK; 3 Biostatistics Department, Institute of Psychiatry, Psychology & Neuroscience at King’s College London, London, UK; 4 King’s Health Economics, Institute of Psychiatry, Psychology & Neuroscience at King’s College London, London, UK; 5 National Centre for Mental Health, Division of Psychological Medicine and Clinical Neurosciences, Cardiff University, London, UK; 6 Division of Psychiatry, University College London, London, UK; 7 Institute of Psychiatry, Psychology & Neuroscience at King’s College London, London, UK; 8 School of Social Work, Care and Community, University of Central Lancashire, Preston, UK; 9 Service User Research Enterprise, Institute of Psychiatry, Psychology & Neuroscience at King’s College London, London, UK; 10 Psychological Medicine and Integrated Care Clinical Academic Group, South London and Maudsley NHS Foundation Trust, London, UK; 11 Greater Manchester Mental Health NHS Foundation Trust, Manchester, UK; 12 Addictions, Institute of Psychiatry, Psychology & Neuroscience at King’s College London, London, UK; 13 Medical and Human Sciences, Institute of Brain Behaviour and Mental Health, Manchester, UK; 14 Manchester Mental Health & Social Care Trust, Manchester, UK

**Keywords:** cohort study, perinatal mental disorder, propensity scoring, cost-effectiveness analysis

## Abstract

**Introduction:**

Research into what constitutes the best and most effective care for women with an acute severe postpartum mental disorder is lacking. The effectiveness and cost-effectiveness of psychiatric mother and baby units (MBUs) has not been investigated systematically and there has been no direct comparison of the outcomes of mothers and infants admitted to these units, compared with those accessing generic acute psychiatric wards or crisis resolution teams (CRTs). Our primary hypothesis is that women with an acute psychiatric disorder, in the first year after giving birth, admitted to MBUs are significantly less likely to be readmitted to acute care (an MBU, CRTs or generic acute ward) in the year following discharge than women admitted to generic acute wards or cared for by CRTs.

**Methods and analysis:**

Quasi-experimental study of women accessing different types of acute psychiatric services in the first year after childbirth. Analysis of the primary outcome will be compared across the three service types, at 1-year postdischarge. Cost-effectiveness will be compared across the three service types, at 1-month and 1-year postdischarge; explored in terms of quality-adjusted life years. Secondary outcomes include unmet needs, service satisfaction, maternal adjustment, quality of mother–infant interaction. Outcomes will be analysed using propensity scoring to account for systematic differences between MBU and non-MBU participants. Analyses will take place separately within strata, defined by the propensity score, and estimates pooled to produce an average treatment effect with weights to account for cohort attrition.

**Ethics and dissemination:**

The study has National Health Service (NHS) Ethics Approval and NHS Trust Research and Development approvals. The study has produced protocols on safeguarding maternal/child welfare. With input from our lived experience group, we have developed a dissemination strategy for academics/policy-makers/public.

**Strengths and limitations of this study**

- Women with lived experience of acute postnatal mental disorders have advocated a study of this kind for a number of years, and a lived experience group informed the development of this study.
- This study will be the first study to provide evidence on the relative effectiveness and cost-effectiveness of mother and baby units, generic acute psychiatric wards and crisis resolution teams.
- A randomised controlled trial study design was not possible due to the large geographical inequity in service provision leading to logistical challenges for randomisation, ethical difficulties in asking women for consent to randomisation when acutely distressed and ill, and strong service preferences of staff, women and families.

## Introduction

Severe postpartum psychiatric disorders are among the most challenging to treat as they are rapid in onset, can deteriorate quickly and are a leading cause of maternal death from suicide.^1^ These disorders may also be associated with deficits in caring for the newborn baby and disruptions in the mother–baby relationship.^1–3^ Over the longer term, the children of mothers admitted to inpatient psychiatric services may develop a range of health, developmental and mental health concerns.^4^ A recent examination of the costs of perinatal mental health problems indicates that the greatest costs relate to adverse child outcomes.^5^

Severe postpartum episodes (which include puerperal psychosis, severe depression or a relapse of bipolar disorder)^1 6^ require acute care which, in most countries, usually means hospital admission,^6^ either in psychiatric mother and baby units (MBUs; available in some parts of Europe, Asia, North America and Australia) or generic inpatient wards. MBUs admit mothers and babies together so that mothers can spend time with their baby as their mental state improves and, potentially, receive help for any difficulties in the mother–infant relationship.^7 8^ Such units are not, however, available everywhere internationally^9^ and data on effectiveness and cost-effectiveness are lacking. In the UK, MBUs are not equally distributed geographically, although this is gradually changing with recent new funding.^10^ The UK National Institute for Health and Care Excellence (NICE) and the Royal College of Psychiatrists recommend that women who develop severe postpartum disorders should be cared for in specialist psychiatric MBUs but evidence to inform commissioning of services on effectiveness and cost-effectiveness is lacking.

Historically, including at the time of recruitment to this study, women in the UK with acute postnatal illnesses could be admitted to generic acute wards, either because access to an MBU was not possible because of geographical distance or a lack of available beds; such admissions necessitate separation from the baby. Alternatively, women could be cared for at home by intensive home treatment teams, also known as crisis resolution teams (CRTs). CRTs care for people who do not require detention under the Mental Health Act but who are experiencing an acute psychiatric episode that would otherwise require hospitalisation. Such teams became available in all National Health Service (NHS) Trusts in England by 2005 and, depending on need, staff can visit service users in their homes daily to avoid acute admission.^11 12^ CRTs treat the mother in her own home and the baby can often remain with her and with her partner or other sources of support.

Research into what constitutes the best and most effective care for women with an acute severe postpartum mental disorder is lacking. The effectiveness and cost-effectiveness of MBUs has not been investigated systematically.^13^ Several studies of MBU admissions describe the clinical and parenting outcomes of mothers^14–20^; two studies report improvements in mother–infant interactions before and after participation in specific mother–infant video feedback interventions,^2 3^ although such before and after designs did not randomise mothers or use a control group comparison. Given limited resources and significant costs of specialist care services, there remains a pressing need for high-quality evidence of the clinical and cost-effectiveness of MBU admission for mothers and infants compared with generic acute wards or CRT services.^13^

Comparison of clinical interventions should be undertaken using a randomised controlled trial design. However, in this population, it is not considered ethical or practical to randomise women, in part because of the large distances many women would need to travel for admission to an MBU leading to logistical challenges for randomisation, ethical difficulties in asking women for consent to randomisation when acutely distressed and ill and because of strong preferences of staff, service users and families.^21^ Therefore, we have designed a quasi-experimental cohort study of women accessing different types of acute psychiatric services within the first year after birth, comparing women’s outcomes to determine clinical effectiveness and cost-effectiveness of MBUs with generic acute wards and CRTs.

## Objectives

Our primary objective is to test the hypothesis that women with an acute psychiatric disorder, in the first year after giving birth, who are admitted to MBUs are significantly less likely to be readmitted to acute care (MBU, CRT or generic acute ward) in the year following discharge from acute care compared with those admitted to generic acute wards or under the care of CRTs.

We also hypothesise that admission to MBUs will be cost-effective compared with admission to generic wards or CRTs for the period between index admission to 1-month postdischarge, and for the period from discharge from index admission to 1-year postdischarge.

We are also testing the following secondary hypotheses:

Women with an acute psychiatric disorder, in the first year after giving birth, admitted to an MBU

- Will have significantly fewer unmet health and social care needs 1-month postdischarge than those admitted to generic acute wards or under CRTs.
- Will report significantly higher levels of service satisfaction 1-month postdischarge than those admitted to generic acute wards or under CRTs.
- Will have better maternal adjustment 1-month postdischarge than those admitted to generic acute wards or under CRTs.
- Will be significantly more sensitive and less unresponsive when interacting with their babies 1-month postdischarge than those admitted to generic acute wards; similarly, their babies will be more cooperative and less passive.
- Will be more likely to retain custody of their child than those admitted to generic acute wards or under CRTs in the year following discharge from acute care.

## Methods

Strengthening the Reporting of Observational Studies in Epidemiology reporting requirements for observational research have been followed (see online supplementary material).

10.1136/bmjopen-2018-025906.supp1 Supplementary data

### Design

The study is a quasi-experimental cohort study embedded within existing service matrices.

### Service definitions

MBUs are defined here as units, with at least four beds and separate from other inpatient units, which provide specialised psychiatric care for both mother and baby where the mother has an acute perinatal psychiatric episode.^21 22^ Acute wards are defined as psychiatric wards that administratively record people receiving care as an inpatient admission, and which provide daily medical cover. CRTs are defined as intensive home treatment mental health teams that manage people in acute crises; the model of care includes rapid response, out of office hours multidisciplinary care.^23^ To ensure CRT treatment is available in study recruitment areas, CRTs have to be able to see people in mental health crises intensively (daily) where necessary; staff have to be available over an extended period (at least 12 hours a day) and the service has to have a specific crisis case load.

### Patient involvement

A lived experience group set up at the time of earlier pilot work (RP-DG-1108–10012) contributed to the writing of the grant proposal and the study design; the primary and secondary outcomes of importance to them; assisted in ensuring that data collection tools were accessible and comprehensible; these tools were then piloted in 21 women.

The lived experience group includes women (and their partners) recruited to represent the broadest possible spectrum of experience of perinatal mental health services. The group includes women who have experienced treatment in psychiatric wards, CRTs, community mental health services and MBUs. This group will continue to meet regularly throughout the duration of the study to advise on the execution and dissemination activities of the study.

### Study sample

Three cohorts of women with acute psychiatric disorders in the first year after childbirth, admitted to psychiatric MBUs, generic acute wards or CRTs are being recruited from mental healthcare provider organisations (Mental Health Trusts in England and Health Boards in Wales), selected to ensure diversity of urbanicity/rurality and access to MBUs. Women are eligible for the study if they used at least one acute service (MBUs, CRTs, generic acute wards), or any combination of all three, during the first year after childbirth.

### Inclusion criteria

- Women with psychiatric disorders needing acute care in the first year after childbirth admitted to psychiatric MBUs, generic acute wards or CRTs.
- Women who have capacity to consent at the point of recruitment (at the point of or after discharge).

There are no diagnostic or language restrictions; we use an interpreter to conduct the research interview in instances where women meet the inclusion criteria but do not speak English.

### Exclusion criteria

- Women using an acute service ‘prophylactically’ (ie, for close monitoring in high-risk cases or for statutory parenting assessments ie, not for acute psychiatric disorder).
- Women whose baby is permanently removed from their care prior to the admission.
- Women without capacity to consent at the point of recruitment.

In order to assess the representativeness of our study sample, we sought to obtain Section 251 approval to collect a minimum dataset— on readmissions, number of inpatient and CRT days, Health of the Nation Outcome Scale, Mental Health Act status and age and ethnicity— from clinical records of all women admitted to each type of service for acute perinatal psychiatric disorders. Unfortunately, our Section 251 application was not approved. It may, however, be possible for us to capture some of this data on the overall MBU population, from national audit data; we are not aware of any such audit data for the other acute care services.

### Recruitment method and study procedures

Our recruitment methods were successfully piloted in a National Institute for Health Research (NIHR) programme development grant (RP-DG-1108–10012) and involve the following: study champions who are clinicians are identified for each ward, MBU and CRT. The study champion’s role is to be the named contact point for routine requests from researchers (every 2–3 weeks) regarding any potentially eligible women admitted to their service. We are also enlisting recruitment support from the regional NIHR Clinical Research Networks (CRNs), using Clinical Studies Officers that are based within trusts to help researchers liaise with acute services to identify eligible women. Engagement of clinicians is key to identifying eligible women and, alongside obtaining CRN support, we are adopting several other strategies to enhance engagement (see box 1: Engagement activities at participating trusts below).

Box 1 **Engagement activities at participating trusts**

- Researchers set up meetings with trust staff to provide presentations on study progress.
- Researchers attend research-specific meetings to keep study champions and other trust staff engaged in the research.
- Researchers send out regular newsletter and social media updates on the study.
- Annual stakeholder workshops are held, providing updates on study progress and continuing professional development lectures from internationally renowned researchers and clinicians in the field of mental health (workshops are free for all study champions to attend).
- Continuing professional development workshops and/or lectures are offered by senior members of the research team.

Postpartum women with capacity (assessed by trust clinical staff), when under acute care or shortly after discharge, are asked to agree or decline to be contacted by a researcher with information about the study. If women agree to be contacted, researchers establish contact with them at the point of, or soon after discharge. Researchers make a maximum of seven attempts to contact each eligible woman about the study (including calls, texts, emails and letters). Women are sent an appropriately worded and formatted participant information sheet and are given at least 24 hours to consider the information before deciding whether to take part. If a woman agrees to participate, the researcher arranges a convenient and safe place to conduct a face-to-face research interview at around 1-month postdischarge. Written informed consent is obtained at the start of the interview, after establishing the woman has capacity to provide informed consent, including asking women to give optional additional consent for researchers to access their case notes to collect information regarding their index admission (ie, baseline data) and their outcomes in the year after discharge, and for a short 5 min telephone interview around 1-year postdischarge to collect follow-up information on what has happened during the study.

Baseline data refers to the time period when women are under the care of acute services (ie, MBUs, CRTs and/or generic acute wards) in the first year after childbirth. Baseline data are collected at the face-to-face research interview and retrospectively from clinical case notes, where women consent to this. Short-term outcome data refers to the time period from discharge from an MBU, CRT or generic acute ward to around 1-month postdischarge. Short-term outcome data are collected at the face-to-face research interview. Long-term outcome data refers to the time period from discharge from an MBU, CRT or generic acute ward to 1-year postdischarge from services. Long-term outcome data are collected from health and social care case notes and via a brief telephone interview with women who have consented to the call. The reason for drawing on health and social care case note/file data is to reduce the potential burden on participants and reduce attrition. See table 1 for a full list of study assessments and data collection time points.

**Table 1:** Outline of measures used for data collection

Mother only measures					
Measure	Details of measure	Data relating to			Hypothesis
		Index admission	One-month postdischarge	One-year postdischarge	
Clinical diagnosis	Case record data on participants’ clinical diagnoses.*	X			
Brief Psychiatric Rating Scale-Expanded	24-item measure that assesses positive, negative and affective symptoms among people with a mental illness^39^; individual item scores are summed, with higher scores indicative of more severe symptomology. For this study, we use case record data for the index episode* and have modified the scoring criteria so that responses are either ‘present’ or ‘absent’, with a score of 1 or 0, respectively.	X			
Mental Health Act detentions	Case record data on Mental Health Act Status*, supplemented by self-report.†	X		X	
Threshold Assessment Grid (TAG), including safeguarding risks to children	7-item scale that assesses the severity of a person’s mental health problems and clinical risk (safety, risk, needs and disabilities).^40^ We modified the TAG to include an additional item on safeguarding risks to children. Scores on the modified TAG range from 0 to 28, with lower scores indicating less severe symptoms.^40^ Ratings of severity of illness (using TAG applied to case notes) are reached by consensus among the research team, to make severity ratings (in the nature subjective) as consistent and reliable as possible.*	X			
Health of the Nation Outcome Scale	Clinician rated scale of health and social functioning of people with severe mental illness in 12 domains (scales)^41^ with each scale rated from 0=‘no problem’ to 4=‘severe to very severe problem’.*	X		X	
Readmissions and CRT service use	Case record data on readmissions to MBUs or generic acute wards and CRT contacts in the year postdischarge*, supplemented by self-report data.†			X	Primary
Drug and alcohol misuse	Case record data on drug and alcohol misuse*, supplemented by self-report data.‡	X			
Safeguarding category of infant	Case record data* and social care data§, supplemented by self-report data‡, on safeguarding status of infant(s) and other children for the index admission and 1-month postdischarge period, and safeguarding status of the index infant at 1-year follow-up.	X	X	X	Secondary
Sociodemographic and clinical factors	Self-report data†‡ and case record data* on sociodemographic/clinical factors, including: age, ethnicity, social class, income, partner status, previous parenting experience, current clinical psychiatric diagnosis, previous psychiatric and medical history (eg, no of acute service contacts in 2 years prior to index admission).	X	X	X	
Modified Pathways to Admission questionnaire	Self-report‡ (supplemented by case record data*) questionnaire of pathways to care following a mental health crisis in the perinatal period,^33^ including service use and contact in the days preceding admission and the circumstances leading up to admission for the current crisis.	X			
Adapted Adult Service Use Schedule (AD-SUS)	Researcher-administered‡ schedule^42^ measures individual-level resource use, including service use by the infant and services related to the birth. It records all-cause hospital and community-based health and social care services, plus mental health related medication use. Specifically, this includes the use of any of the following for the women and/or her index baby: accommodation (provided by the NHS or Local Authorities), services for looked after children (fostering, adoption, formal kinship, etc), inpatient stays, outpatient appointments, day patient contacts, accident and emergency contacts, community health and social care contacts and mental health related medication. The AD-SUS covers the period of the index admission to 1-month postdischarge period. The adapted AD-SUS was piloted in the relevant population as part of the Effectiveness and cost-effectiveness of Mother and Baby Units versus general psychiatric inpatient wards and Crisis Resolution Team services (ESMI) study National Institute for Health Research programme development grant (RP-DG-1108–10012).		X		Primary
Camberwell Assessment of Need for Mothers (short version)	Researcher-administered‡ 26-item questionnaire that assesses the health and social care needs for mothers with severe mental illness,^43^ scored on a scale of either 1=‘met need’, 2=‘unmet need’ or 0=‘no problem’; the sum of the ‘met need’ and ‘unmet need’ items generate a total score of the number of needs.	X	X		Secondary
Modified Composite Abuse Scale	Self-report‡ 30-item questionnaire assessing experiences of partner abuse.^44^ Items are rated from 0=‘never’ to 5=‘daily’, with total scores ranging from 0 to 150. A cut-off point of 3 is assigned, with scores of 3 or more indicating partner violence. The scale has been modified for this study to collect data covering the two time periods: (1) prior to admission and (2) since discharge.	X	X		
Modified Social Provisions Scale	Researcher-administered‡ 24-item questionnaire that assesses the degree to which an individual’s social relationships provide various dimensions of social support.^45^ Items are rated on a four-point Likert scale ranging from 1=‘strongly disagree’ to 4=‘strongly agree’.^45^ 6 domains of social provision (ie, guidance, reassurance of worth, social integration, attachment, nurturance and reliable alliance) are calculated by summing the scores of specific items on the questionnaire; high scores on each of the domains indicate that the person is receiving that social provision. The scale has been modified to collect data covering the two time periods of interest for the study: (1) prior to admission and (2) since discharge.	X	X		
Short Form 36 Health Survey	Self-report‡ 36-item questionnaire which produces a preference-based single index measure of general health.^46^ It measures health on eight multi-item dimensions: (1) limitations in physical activities because of health problems; (2) limitations in social activities because of physical or emotional problems; (3) limitations in usual role activities because of physical health problems; (4) bodily pain; (5) general mental health (psychological distress and well-being); (6) limitations in usual role activities because of emotional problems; (7) vitality (energy and fatigue) and (8) general health perceptions.^47^ This measure can be used to calculate quality-adjusted life years (QALYs).		X		Primary
EuroQol five-dimension scale (EQ-5D-5L)	Self-report†‡ preference-based measure of health-related quality of life measured on five dimensions (mobility, self-care, usual activities, pain/discomfort and anxiety/depression), each rated on five levels (no problems, slight problems, moderate problems, severe problems and extreme problems).^48^ This measure can be used to calculate QALYs.		X	X	Primary
Perinatal VOICE questionnaire	27-item self-report‡ questionnaire that examines admission processes and therapeutic activities on wards. It contains six sections relating to experience of care on admission: (1) care and treatment (three items); (2) medication (two items); (3) staffing (seven items); (4) environment (five items) and (5) baby’s well-being (10 items). At the end of each section, respondents are encouraged to provide further comments about their experience of care.		X		Secondary
Client Satisfaction Questionnaire	Self-report‡ questionnaire^49^ of experiences of health service use. Eight items are rated on a four-point scale and explore degrees of service satisfaction (eg, ‘how would you rate the quality of service you received?’ ‘How satisfied are you with the amount of help you received?’); higher scores indicate greater satisfaction. We asked a further two items to assess the satisfaction of care in relation to the baby (‘How satisfied were you with the advice you received about your baby from the service?’) and a free-text feedback item on the most and least helpful aspects of service use (‘what were the most/least helpful aspects of the service?’).		X		Secondary
The Postpartum Bonding Questionnaire	Self-report‡ 25-item questionnaire designed to provide an early indication of disorders within mother–infant relationships, through the assessment of a mother’s feelings and attitudes towards her infant.^50^ Individual items are rated on a six-point scale (0–5) and are used to obtain four subscale scores: (1) impaired bonding (12 items, scores ranging from 0 to 60), (2) rejection and anger (seven items, scores ranging from 0 to 35), (3) anxiety about care (four items, scores ranging from 0 to 20) and (4) risk of abuse (two items, scores ranging from 0 to 10); higher scores indicate increased difficulties.		X		Secondary
Childhood Trauma Questionnaire	Self-report‡ 28-item questionnaire designed to assess five types of negative childhood experiences: (1) emotional neglect, (2) emotional abuse, (3) physical neglect, (4) physical abuse, and (5) sexual abuse.^51^ Items are rated on a five-point scale (–5), from ‘never true’ to ‘very often true’, with scores ranging from 5 to 25 for each of the abuse types.		X		
Mother/infant measures					
Mother–infant interactions	Mother–infant interactions are captured in a 3 min video clip taken during play at home‡ and subsequently assessed by a trained rater, unaware of participant service use, using the Child and Adult Relational Experimental Index.^52^ Coding of the interaction takes between 15 and 20 min and focuses on seven aspects of both mother and infant behaviour: (1) affection; (2) body contact; (3) facial expression; (4) verbal expression; (5) turn-taking; (6) control and (7) developmental appropriateness of chosen activity). Each aspect of mother and infant behaviour is evaluated individually and summed to make seven scale scores, with each scale scored on a range from 0 to 14. For mothers these scales are sensitivity, control and unresponsiveness and for infants these scales are cooperativeness, compulsivity, difficultness and passivity. Higher scores of control and unresponsiveness on the mother scales and higher scores of compulsivity, difficultness and passivity on the infant scales are indicative of poorer dyadic synchrony. Higher scores of sensitivity on the mother scale and higher scores of cooperativeness on the infant scale are indicative of stronger dyadic synchrony		X		Secondary
Bayley Scales of Infant Development	Researcher-administered scales‡ that examine motor (fine and gross), language (receptive and expressive) and cognitive development of infants and toddlers.^53^		X		
Infant growth trajectories	Case note data on infants APGAR scores and early weight measurements‡	X			

*indicates that these data are extracted from clinical case records.

†indicates that these data are collected at a 1-year follow-up telephone interview.

‡indicates that these data are collected at a 1-month postdischarge face-to-face interview.

§indicates that these data are collected from social care records.

CRT, crisis resolution team; MBUs, mother and baby units; NHS, National Health Service.

The face-to-face research interview—conducted around a month after women have been discharged from acute services—takes up to 3 hours and includes assessment of outcomes for both women and their infants. Home visits are offered to women (where deemed safe and appropriate by clinical teams) so that women do not need to travel to interviews, and they can complete the interview over one or two sessions depending on their preference. Women with older children who require childcare to take part are reimbursed for the costs of childcare. Interpreters are used for women who do not speak the same language as the researcher and who wish to take part. Women are offered up to £25 worth of vouchers as a thank you for taking part; women receive £15 of vouchers for completing assessments about themselves and an additional £10 for also completing the baby assessments.

During the face-to-face interview, participants are asked to nominate a significant other (eg, partner, family member and close friend) who supported them during their time under acute services. If a significant other is identified and consents, he/she is asked to complete a short 10–15 min questionnaire about his/her relationship with the participant, his/her experiences of caregiving for the participant and its impact, and items assessing current distress and mental health status. To facilitate data collection from significant others, the researchers offer significant others the chance to complete the questionnaires online or in paper format, with prepaid envelopes provided. Successful returnees receive £10 shopping vouchers as a thank you for their contribution.

**Figure 1:**
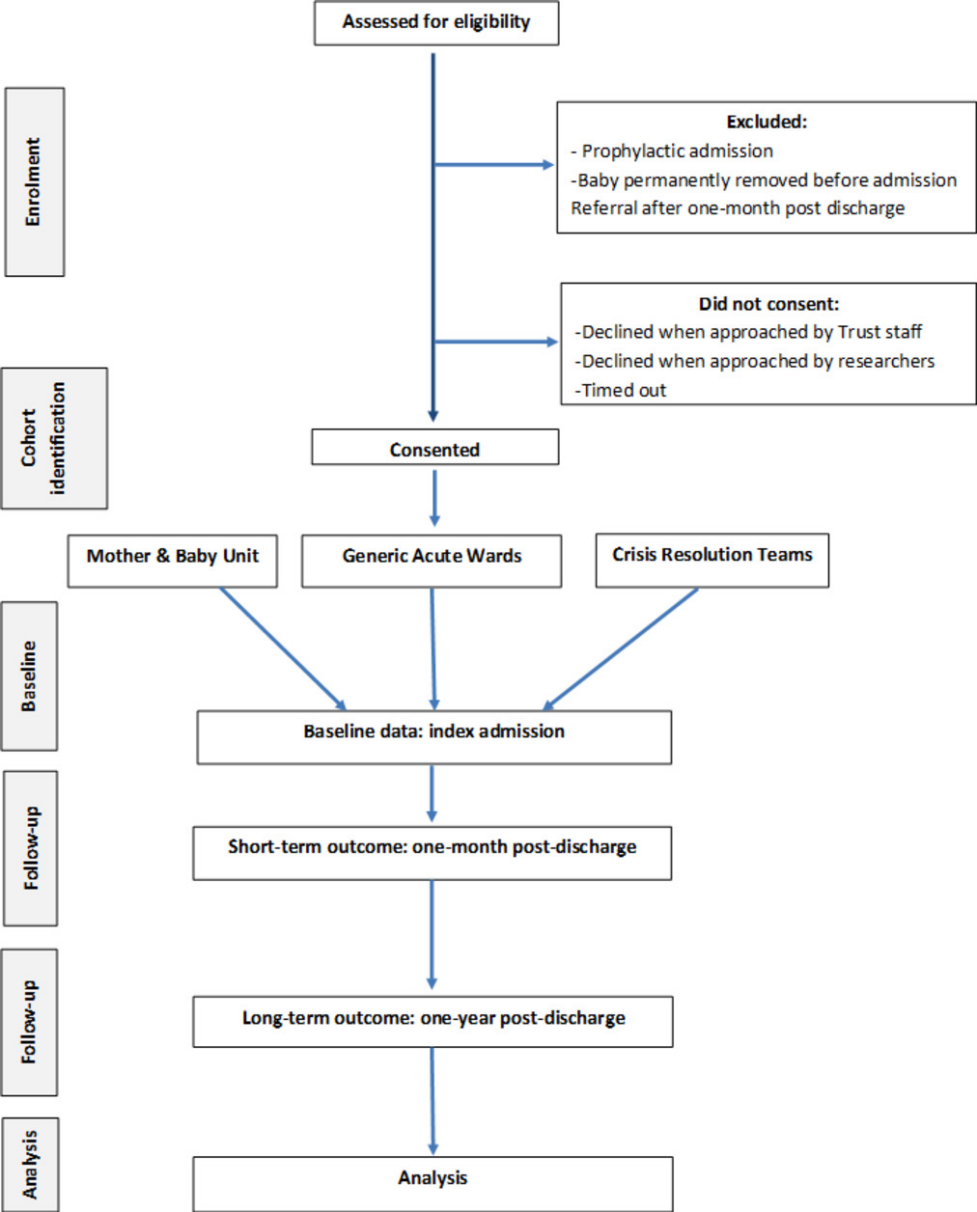
Flow of participant diagram.

See image figure 1: Flow of participant diagram which illustrates the flow of participants through the study, based on the Consolidated Standards of Reporting Trials diagram^24^ and the Quality of Reporting of Observational Longitudinal Research guidelines.^25^

### Measures

Table 1 outlines the measures used to collect data at all study time points, for women and their infants.

### Process evaluation

As service provision varies nationally, we are collecting detailed descriptions of the service components of MBUs, CRTs and generic acute wards in participating provider organisations. We have developed a structured Process Evaluation questionnaire (available on request) guided by the research literature and via discussions between researchers and Programme Management Group members. The questionnaire is structured around three service component types: interventions, facilities and staff.

The ‘interventions’ component of the questionnaire comprises four distinct categories which examine the availability of specific types of interventions in the service: (1) psychological, (2) infant–parent relationship support, (3) support for partners/significant others and (4) social support. To chart exactly how these interventions are delivered, the questionnaire distinguishes between whether the services directly provide the intervention (ie, by staff within MBU/acute ward/CRT) or indirectly provide the intervention (ie, by other staff usually from external organisations, eg, health visitor). The questionnaire also charts the frequency in which interventions are delivered, defined as either: (1) ‘routinely provided’ (ie, a standard part of care received by many women) or (2) ‘occasionally provided’ (ie, provided on an ad hoc basis).

The ‘facilities’ and ‘staff’ components of the questionnaire capture whether services have: (1) ‘full access’ to facilities and staff (ie, facilities are included within the main service; staff are part of the core service team); (2) ‘partial access’ to facilities and staff (eg, psychologist providing CBT but not within the index unit) or (3) ‘no access’ to facilities and staff. The questionnaire has been piloted, and adjustments made where necessary.

Administration of the questionnaire is undertaken by a member of the research team, who schedules a telephone contact with a senior member of each service type and completes the questionnaire over the telephone. The questionnaire is emailed to the staff member ahead of the structured telephone interview, so they can review the forms and prepare answers in order to facilitate ease of completion.

### Power calculation

Our pilot data using the Clinical Record Interactive Search (CRIS) database (the anonymised Case Register^26^ local to King’s College Hospital, London) revealed the following for 20 perinatal women on generic acute wards, 20 admitted to MBUs and 20 under CRTs: acute ward patients were most likely to be readmitted to these services with 95% being readmitted at some point during 12-month follow-up, compared with 35% of women who received MBU care; CRT readmission rates were similar to MBUs. Therefore, assuming similar readmission rates nationally for MBU patients (35%), we could detect a doubling of risk for generic acute ward patients with 90% power with 47 women in each group. We aim to recruit 100 women in each group; to allow for 20% attrition and exclusion of women for being beyond the ‘region of support’ (ie, whose characteristics make them unmatchable with women in another treatment arm—see Statistical methods section for further details).

## Analysis

### Statistical methods

There is limited availability of specialist MBU beds and it is, therefore, likely that some women who would be offered an MBU bed at first presentation are admitted to an acute ward, while waiting for a bed. As some women may also receive care from more than one type of inpatient service (ie, MBU and acute ward) during their index admission, in our main analysis MBUs will be classified as the ‘highest level of care’ and we will run two sensitivity analyses—one based the majority of days spent within a specific inpatient service and the other based on first service accessed.

We shall use a propensity score approach to account for systematic differences between MBU and non-MBU participants. This approach allows the exact specification of the covariate adjustment to be determined blind to the outcome data, thereby reducing the risk of unintended bias. The Stata command p score will estimate the propensity score of the treatment (MBU or non-MBU) on specified covariates, selected using problem knowledge and exploratory comparison of cohorts, using a probit model and will stratify individuals in blocks according to the propensity score. The blocks are determined by a balancing algorithm and the balancing property within each block is tested, to ensure sufficient blocks are used to adequately balance the covariates. We will also remove women with characteristics that place them beyond the ‘region of support’ and thus for whom there are no ‘matches’, that is, women with propensity scores either so high or so low that there are insufficient similar women receiving either MBU or non-MBU treatment to make a comparison; we will report characteristics and outcomes for these women separately.

Once the propensity scores have been formed, they will be included in the primary and secondary analyses through use of the inverse propensity score weights^27^ (ie, weighted regressions will be performed), which will also be combined with the inverse probability weights for drop-out, known as attrition weights, specific to each endpoint measure. The readmission rate will be modelled using logistic regression, with adjustment for baseline measures that are likely to increase power, that is, baseline measures of outcome and symptom severity. Point estimates, CI and significant tests based on the sandwich estimator of the parameter covariance matrix^28^ will be reported. Analysis on the primary outcome will include stratum specific treatment estimates. Trends in effect estimate over strata can be informative as to variability of effect, an advantage of using the stratification matching technique. The computation of average treatment effects will be restricted to the common region of support. Sensitivity analyses will also be performed.

We shall also examine geographical and temporal variation in MBU services as a source of instrumental variables to account for unmeasured selection effects.

Missing data will be accounted for on three levels: single imputation or prorating for sporadic missing item-level data that contribute to scores, multiple imputation for entirely missing scales or factors and listwise deletion for those with insufficient data to allow plausible imputation.

Sensitivity analyses will examine the impact of different treatment arm definitions, formed following an examination of the observed treatment pathway patterns.

### Economic evaluation

We will evaluate whether MBU services are cost-effective in the short term (from index admission to 1-month postdischarge) and the longer term (from discharge from the index admission to 1-year postdischarge) in the treatment of women with severe mental illness following birth, compared with generic acute wards and CRTs. Analysis from admission to 12 months was not considered appropriate because lengths of index admission may vary greatly, which would heavily influence the total cost and thus the results of the cost-effectiveness analysis.

The economic evaluation at 1-month postdischarge will take the NHS and personal social services perspective preferred by NICE,^29^ with data collected in face-to-face interviews with participants using the Adult Service Use Schedule (AD-SUS) (see table 1 for full details of the AD-SUS measure). Since the index admission/acute care is the intervention, and since the development work indicated that this can be difficult for women to recall, data on this will be taken from clinical notes. The AD-SUS, therefore, focuses primarily on hospital and community-based contacts postdischarge from the index admission.

The economic evaluation at 1-year postdischarge will take a narrower mental health perspective, with mental health service use data collected from clinical records and the EuroQol five-dimension scale (EQ-5D-5L) collected via brief telephone interviews, given no face-to-face interviews with participants will take place at this time point. Resource use data for the period from the date of discharge from the index admission to the 1-year postdischarge follow-up will be collated using a proforma created by the research team and collected from secondary mental health records. The proforma was piloted and edited as needed to ease data completion. This proforma will include all contacts with secondary mental health services including further periods in MBU, generic acute ward or CRT care plus any outpatient or community mental health contacts. A briefer version of the proforma will be used to collect data on the use of key acute services (MBUs, generic acute wards or CRTs) in the 2-year period prior to the index admission.

Resource use data will be combined with unit costs from national published sources to calculate the total cost of participants admitted to the MBU, CRT and generic acute wards for the two different time periods (from index admission to 1-month postdischarge and from discharge from the index admission to 1-year postdischarge).

Costs and outcomes will be compared and presented in terms of mean differences and 95% CIs obtained by non-parametric bootstrap regressions (10 000 replications) to account for the non-normal distribution commonly found in economic data. Cost-effectiveness will be assessed through the calculation of incremental cost-effectiveness ratios^30^ and will be explored in terms of quality-adjusted life years (QALYs) calculated from the EQ-5D-5L^31^ and using the area under the curve approach.^32^ Utility data are not being collected at baseline as women are not being interviewed until after discharge from the index admission. We will, therefore, use published utility values for a similar population (women in crisis (eg, Howard *et al* 33)) as a substitute for utility at baseline. To provide more relevant treatment-effect estimates,^34^ all cost-effectiveness analyses will include prespecified covariates, in line with the main clinical analysis, plus the baseline variable of interest, where available. Uncertainty around the cost and effectiveness estimates will be represented by cost-effectiveness acceptability curves.^35^ The Short-Form Six-Dimension will be available at 1-month postdischarge and will be used to calculate QALYs to explore cost-effectiveness in the short term in a sensitivity analysis.^36^ Sensitivity analyses will also explore the impact of missing data.

## Ethics

The study has obtained NHS Research Ethics Committee approval from the London-Camberwell St. Giles committee (number: 14/LO/0765). Research and development (R&D) approval has been granted by all participating trusts and the three Welsh Health Boards, via the Wales Health and Care Research Permissions service. All researchers complete relevant training - for example, NIHR Good Clinical Practice in Secondary Care and the Health and Social Care Information Centre Information Governance training; assessment of mental capacity; extraction of case note data —to ensure that they are appropriately skilled to undertake research with study participants.

Some participants will be very vulnerable, and our team will ensure close supervision of researchers to safeguard maternal and child welfare. Detailed guidance on safeguarding maternal and child welfare are outlined in the programme standard operating procedures,^37^ including instructions that researchers should follow if they identify any safeguarding concerns (ie, researchers will first contact one of the senior experienced clinical applicants on the programme grant to discuss their concerns and to identify what actions to undertake). Researchers undertake regular clinical supervision with a senior clinician who is part of the research team.

Safety protocols have also been developed to ensure that participants, their families and researchers remain safe when making contact, conducting research and afterwards. This includes the following precautions:

- On initial contact, researchers establish an appropriate contact number and time for future contact between themselves and participants.
- Researchers will ensure that the location(s) where an interview takes place is private and secure and cannot be overheard. The information provided by participants will be confidential and anonymised. In some situations, however, it may be necessary to disclose personal information without a patient’s consent if it is in the public interest (ie, where a failure to do so may expose the patient or others at risk of death or serious harm). The limits of confidentiality are explained on the participant information sheet and will be discussed with all participants as part of the informed consent process. The General Medical Council guidance on confidentiality will be followed.^38^ The researchers will contact one of the clinical applicants on the grant to discuss any situations when confidentiality may need to be broken.
- After the interview, researchers will ask participants how they feel and if they would like to discuss anything further with their responsible clinician.
- Researchers will give details of interview locations, start times and approximate end times to colleagues at their research department.

A study-specific protocol has been developed for the appropriate handling, management, storage and transfer of data. All data are stored in accordance with the Data Protection Act (2018) and General Data Protection Regulations, with which all members of the research team are familiar.

## Dissemination

This study will provide evidence on effectiveness and cost-effectiveness (reducing readmission rates and improving quality of life), as well as identifying the services with which women themselves are most satisfied and those which produce the best outcomes for mothers (functioning, met needs), their infants (quality of mother–infant interaction) and significant others around them (carers’ needs). Throughout the study, we will produce regular newsletters and electronic updates for professionals and lay stakeholders. We will also hold yearly perinatal mental health workshops throughout the study to showcase the work to stakeholders and service users. We will present the study as poster and/or oral presentations at national and international conferences; via social media (eg, Twitter) and press releases (eg, Maternal Mental Health Alliance); via articles for professionals, policy-makers and the public; via video talks and podcasts to relevant Royal Colleges, non-governmental organisations and public groups. The main study findings will be published in an open access peer-reviewed journal, and we will highlight the work in relevant resource lists (eg, Public Health England Perinatal and Infant Mental Health eBulletin, Child and Maternal Health Knowledge Update of the National Child and Maternal Health Intelligence Network).

### Study status

Recruitment of women admitted to services up to 31 December 2017 was completed on 6 March 2018. Data collection of primary outcomes will continue until spring 2019.

## Supplementary Material

Reviewer comments

Author's manuscript
