# Supplementary material for: Protocol for a quasi-experimental study of the effectiveness and cost-effectiveness of mother and baby units compared with general psychiatric inpatient wards and crisis resolution team services (The ESMI study) in the provision of care for women in the postpartum period
Source: BMJ Open. 2019 Mar 23;9(3):e025906. doi: 10.1136/bmjopen-2018-025906 (PMC6475160; doi:10.1136/bmjopen-2018-025906)
Supplement: Supplementary data [file bmjopen-2018-025906supp001.pdf]

STROBE 2007 (v4) Statement—Checklist of items that should be included in reports of *cohort studies*

Section/Topic	Item #	Recommendation	Reported on page #
**Title and abstract**	1	(*a*) Indicate the study's design with a commonly used term in the title or the abstract	Pg 1 and pg 6
		(*b*) Provide in the abstract an informative and balanced summary of what was done and what was found	Pg 3
**Introduction**			
Background/rationale	2	Explain the scientific background and rationale for the investigation being reported	Pg 4-5
Objectives	3	State specific objectives, including any prespecified hypotheses	Pg 6
**Methods**			
Study design	4	Present key elements of study design early in the paper	Pg 6-7
Setting	5	Describe the setting, locations, and relevant dates, including periods of recruitment, exposure, follow-up, and data collection	Pg 7-10 and pg 21-22
Participants	6	(*a*) Give the eligibility criteria, and the sources and methods of selection of participants. Describe methods of follow-up	Pg 7-10 and Figure 1
		(*b*) For matched studies, give matching criteria and number of exposed and unexposed	n/a
Variables	7	Clearly define all outcomes, exposures, predictors, potential confounders, and effect modifiers. Give diagnostic criteria, if applicable	Pg 12-16
Data sources/ measurement	8*	For each variable of interest, give sources of data and details of methods of assessment (measurement). Describe comparability of assessment methods if there is more than one group	Pg 12-16
Bias	9	Describe any efforts to address potential sources of bias	Pg 8 and pg 18-20
Study size	10	Explain how the study size was arrived at	Pg 17-18
Quantitative variables	11	Explain how quantitative variables were handled in the analyses. If applicable, describe which groupings were chosen and why	Pg 18-20
Statistical methods	12	(*a*) Describe all statistical methods, including those used to control for confounding	Pg 18-20
		(*b*) Describe any methods used to examine subgroups and interactions	Pg 18-20
		(*c*) Explain how missing data were addressed	Pg 18-20
		(*d*) If applicable, explain how loss to follow-up was addressed	
		(*e*) Describe any sensitivity analyses	Pg 18-20
**Results**			

For peer review only

Participants	13*	(a) Report numbers of individuals at each stage of study—eg numbers potentially eligible, examined for eligibility, confirmed eligible, included in the study, completing follow-up, and analysed	n/a
		(b) Give reasons for non-participation at each stage	n/a
		(c) Consider use of a flow diagram	Figure 1
Descriptive data	14*	(a) Give characteristics of study participants (eg demographic, clinical, social) and information on exposures and potential confounders	n/a
		(b) Indicate number of participants with missing data for each variable of interest	n/a
		(c) Summarise follow-up time (eg, average and total amount)	n/a
Outcome data	15*	Report numbers of outcome events or summary measures over time	n/a
Main results	16	(*a*) Give unadjusted estimates and, if applicable, confounder-adjusted estimates and their precision (eg, 95% confidence interval). Make clear which confounders were adjusted for and why they were included	n/a
		(*b*) Report category boundaries when continuous variables were categorized	n/a
		(*c*) If relevant, consider translating estimates of relative risk into absolute risk for a meaningful time period	n/a
Other analyses	17	Report other analyses done—eg analyses of subgroups and interactions, and sensitivity analyses	n/a
**Discussion**			
Key results	18	Summarise key results with reference to study objectives	n/a
**Limitations**			
Interpretation	20	Give a cautious overall interpretation of results considering objectives, limitations, multiplicity of analyses, results from similar studies, and other relevant evidence	n/a
Generalisability	21	Discuss the generalisability (external validity) of the study results	n/a
**Other information**			
Funding	22	Give the source of funding and the role of the funders for the present study and, if applicable, for the original study on which the present article is based	Pg 2

*Give information separately for cases and controls in case-control studies and, if applicable, for exposed and unexposed groups in cohort and cross-sectional studies.

Note: An Explanation and Elaboration article discusses each checklist item and gives methodological background and published examples of transparent reporting. The STROBE checklist is best used in conjunction with this article (freely available on the Web sites of PLoS Medicine at http://www.plosmedicine.org/, Annals of Internal Medicine at http://www.annals.org/, and Epidemiology at http://www.epidem.com/). Information on the STROBE Initiative is available at www.strobe-statement.org.

For peer review only
