# Supplementary material for: Protocol for a quasi-experimental study of the effectiveness and cost-effectiveness of mother and baby units compared with general psychiatric inpatient wards and crisis resolution team services (The ESMI study) in the provision of care for women in the postpartum period
Source: BMJ Open. 2019 Mar 23;9(3):e025906. doi: 10.1136/bmjopen-2018-025906 (PMC6475160; doi:10.1136/bmjopen-2018-025906)
Supplement: Author's manuscript [file bmjopen-2018-025906.draft_revisions.pdf]

PEER REVIEW HISTORY

BMJ Open publishes all reviews undertaken for accepted manuscripts. Reviewers are asked to complete a checklist review form (**http://bmjopen.bmj.com/site/about/resources/checklist.pdf**) and are provided with free text boxes to elaborate on their assessment. These free text comments are reproduced below.

ARTICLE DETAILS

**TITLE (PROVISIONAL)**	A quasi-experimental study of the Effectiveness and cost-effectivenesS of Mother and Baby Units compared with general psychiatric Inpatient wards and crisis resolution team services (The ESMI study) in the provision of care for women in the post-partum period
**AUTHORS**	Trevillion, Kylee; Shallcross, Rebekah; Ryan, Elizabeth; Heslin, Margaret; Pickles, Andrew; Byford, Sarah; Jones, I; Johnson, Sonia; Pawlby, Susan; Stanley, Nicky; Rose, Diana; Seneviratne, Gertrude; Wieck, Angelika; Jennings, Stacey; Potts, Laura; Abel, Kathryn; Howard, Louise

VERSION 1 - REVIEW

**REVIEWER**	Tanya Wright<br>University of Auckland, New Zealand
**REVIEW RETURNED**	20-Aug-2018

**GENERAL COMMENTS**	This manuscript addresses an important issue- the real-world utilisation, patient experience, clinical- and cost-effectiveness of inpatient and acute services for women with major mental illness and their infants. Three different service configurations are compared. The findings are likely to contribute to health service planning internationally and hence the health outcomes for women, children and families. The outcomes measured are important, multi-faceted and will be of interest to clinicians, researchers and commissioners of services.<br>This is a very well- written manuscript and proposal, which provides appropriate study design detail, data collection and analysis methodology. It is ethically and procedurally sound.<br>The research team are commended on their inclusion of contribution from people with lived experience from the outset of the project and in aspects of the study design. It is a strength of the study that there is inclusion of non-English speakers and that explicit and extensive engagement strategies for recruitment are described. The development of a Process Evaluation questionnaire is also very valuable as clinical contexts are complex and in general insufficiently explained.<br>A limitation is that the follow-up after hospital admission is short. However this is necessarily so, as this is operational research in an active field of enquiry. However, the infants of mothers with mental illness of a severity warranting hospitalisation are in jeopardy as well, with demonstrable health, developmental and mental health concerns of their own (Wright, Stevens, & Wouldes, 2018). The greatest societal burden and long-term cost is due to child outcomes (Bauer, Parsonage, Knapp, Iemmi, & Adelaja,

	2014), so these are important to acknowledge. This study will open the door for further longitudinal analysis.<br>I have only one suggestion, which pertains to addressing selection bias. The most unwell and "hard to reach" women and infants may not be well represented. This arises as a consequence of competence to consent being determined at discharge, the large number of self-reported questionaires, propensity score matching, dropout and listwise deletion for those with insufficient data for imputation. Is it possible to include a parameter to assess if participants of the study are representative of the base population?<br><br>References<br>Bauer, A., Parsonage, M., Knapp, M., Iemmi, V., & Adelaja, B. (2014). The costs of perinatal mental health problems. London: Centre for Mental Health and London School of Economics.<br>Wright, T., Stevens, S., & Wouldes, T. A. (2018). Mothers and their infants co-admitted to a newly developed mother–baby unit: Characteristics and outcomes. Infant Mental Health Journal, 36(6), in press.

**REVIEWER**	Florence Gressier<br>Inserm UMR1178, CESP, Univ Paris Sud, Department of Psychiatry, Hôpital de Bicêtre, Le Kremlin Bicêtre, France
**REVIEW RETURNED**	26-Aug-2018

**GENERAL COMMENTS**	"A quasi-experimental study of the Effectiveness and cost-effectiveness of Mother and Baby Units compared with general psychiatric Inpatient wards and crisis resolution team services (The ESMI study)"<br>The protocol is scientifically credible and is presented in an appropriate context ; the design is ethically and procedurally sound. The protocol reports an ongoing study.<br><br>The primary aim is to investigate the effectiveness and cost-effectiveness of psychiatric Mother and Baby Units (MBUs).<br>The primary hypothesis is that women with an acute psychiatric disorder, in the first year after giving birth, admitted to MBUs are significantly less likely to be readmitted to acute care (an MBU, Crisis Resolution Teams CRTs or generic acute ward) in the year following discharge than women admitted to generic acute wards or cared for by CRTs.<br><br>The protocol study is well structured and written. This is an essential study for perinatal mental health and MBUs.<br><br>Minor remark: In title, the authors should mention the post-partum period.

VERSION 1 – AUTHOR RESPONSE

Reviewer one

We thank reviewer one for the positive comments and for the detailed review of the paper. We have now included a reference to the two papers provided by the reviewer within the Introduction, page 4, paragraph 4:

"Severe postpartum psychiatric disorders are among the most challenging to treat as they are rapid in onset, can deteriorate quickly and are a leading cause of maternal death from suicide 1. These disorders may also be associated with deficits in caring for the new-born baby and disruptions in the mother-baby relationship 1-3. Over the longer-term, the children of mothers admitted to inpatient psychiatric services may develop a range of health, developmental and mental health concerns 4. A recent examination of the costs of perinatal mental health problems indicates that the greatest costs relate adverse child outcomes 5."

Reviewer one also raised an important point regarding the possibility of including a parameter to assess if study participants are representative of the base population. This issue is something that the study team have sought to address from the outset of the study. We attempted to obtain Section 251 approval to gather some key demographic and clinical data on the overall base population, in order to use as a comparison with our sample, but unfortunately our application was not approved. The team have carefully considered alternative options to gathering this information and believe it may be possible to capture such data on the MBU base population, from national audit data. Unfortunately, we are not aware of any such audit data for the other acute care services. The team will seek to request the MBU audit data from the relevant organisations. We have now made reference to this point in the manuscript, on page 8, paragraph three:

“In order to assess the representativeness of our study sample, we sought to obtain Section 251 approval to collect a minimum dataset - on readmissions, number of inpatient and CRT days, HONOS, Mental Health Act status and age and ethnicity - from clinical records of all women admitted to each type of service for acute perinatal psychiatric disorders. Unfortunately, our Section 251 application was not approved. It may, however, be possible for us to capture some of this data on the overall MBU population, from national audit data; we are not aware of any such audit data for the other acute care services”

Reviewer two

We thank reviewer two for the positive comments. We have addressed their point about the revision to the protocol title, and we now include a mention of the post-partum period. The title has now been changed as follows:

"A quasi-experimental study of the Effectiveness and cost-effectivenesS of Mother and Baby Units compared with general psychiatric Inpatient wards and crisis resolution team services (The ESMI study) in the provision of care for women in the post-partum period"
